# Measurement of serum 1,5-AG provides insights for diabetes management and the anti-viral immune response

**DOI:** 10.1007/s00018-024-05568-7

**Published:** 2025-02-06

**Authors:** Marcus Tong Zhen Wei, Linda A. Gallo, Katina D. Hulme, Fawaz Alzaid, Jean-Baptiste Julla, Emily S. Dorey, Gilles Morineau, Keng Yih Chew, Emma J. Grant, Stephanie Gras, Helen L. Barett, Jean-Pierre Riveline, Meagan Carney, Kirsty R. Short

**Affiliations:** 1 School of Chemistry and Molecular Biosciences, The University of Queensland, St Lucia, Australia; 2 School of Health, University of the Sunshine Coast, Petrie, Australia; 3 Department of Medical Microbiology, Academic Medical Center, University of Amsterdam, Amsterdam, Netherlands; 4 Université Paris Cité, CNRS, INSERM, Institut Necker Enfants Malades-INEM, Paris, F-75015 France; 5 Dasman Diabetes Institute, Kuwait City, Kuwait; 6 Department of Diabetes, Lariboisière Hospital, Assistance Publique - Hôpitaux de Paris And Paris-Cité University, Paris, France; 7 Mater Research, The University of Queensland, South Brisbane, QLD 4101 Australia; 8 Department of Biochemistry and Molecular Biology - GHU AP- HP.Nord, Université Paris Cité, Lariboisière Hospital, Paris, France; 9 Infection and Immunity Program, La Trobe Institute for Molecular Science (LIMS), La Trobe University, Bundoora, VIC 3086 Australia; 10 Department of Biochemistry and Chemistry, School of Agriculture, Biomedicine and Environment (SABE), La Trobe University, Bundoora, VIC 3086 Australia; 11 Department of Biochemistry and Molecular Biology, Monash University, Clayton, VIC Australia; 12 University of New South Wales Medicine, Kensington, Australia; 13 Obstetric Medicine, Royal Hospital for Women, Randwick, Australia; 14 School of Mathematics and Physics, The University of Queensland, St Lucia, Australia; 15 Australia Infectious Diseases Research Centre, The University of Queensland, St Lucia, Australia; 16 Queensland Immunology Research Centre, The University of Queensland, St Lucia, Australia

**Keywords:** Glycaemic variability, 1-5-AG, HbA1c, Immune response

## Abstract

**Background:**

Achieving an in-range glycated haemoglobin (HbA1c) is essential for managing diabetes mellitus (DM). However, this parameter provides an estimate of long-term blood glucose control rather than daily glycaemic variations. Glycaemic variability can be more predictive than HbA1c in terms of identifying those at risk for diabetes complications, including risk of severe respiratory virus infections and is usually measured via a continuous glucose monitor (CGM). For individuals for whom a CGM is not available, serum 1,5 anhydroglucitol (1,5-AG) level has shown potential as an alternative method for monitoring glycaemic variability. Despite this, at present 1,5-AG is not routinely used in the clinical assessment of DM. Here, we aim to determine whether assessing 1,5-AG, in addition to HbA1c, is of any potential clinical utility to the management of DM for patients.

**Methods:**

Using machine learning and data derived from 78 patients with type I DM (for whom CGM data is available) we show that the combination of 1,5-AG and HbA1c improves the prediction of a patient’s glycemia risk index (GRI) compared to HbA1c alone.

**Results:**

The GRI is an essential tool in the management of DM as it reflects both clinical priorities and patient centred outcomes. The inclusion of 1,5-AG in this prediction was particularly important for individuals who had very high or very low GRI. Furthermore, in the context of glycaemic variability and susceptibility to severe respiratory virus infections, we show that reduced 1,5-AG in the plasma is associated with reduced ex vivo CD4 + T cell cytokine responses to influenza virus in individuals with a matched HbA1c.

**Conclusions:**

Taken together, these data argue for an increased monitoring of 1,5-AG in the clinic for individuals without a CGM to provide additional insights for diabetes management.

**Supplementary Information:**

The online version contains supplementary material available at 10.1007/s00018-024-05568-7.

## Introduction

Achieving an in-range glycated haemoglobin (HbA1c) is a key clinical target of diabetes mellitus (DM) management. The average lifespan of red blood cells is approximately 2–3 months, meaning that HbA1c provides an estimate of long-term blood glucose control, rather than glycaemic variability [1]. Glycaemic variability refers to peaks and troughs in blood glucose levels that may occur in people with diabetes both within the day and across different days [1]. Importantly, two individuals can have the same HbA1c but marked differences in their glycaemic variability [1]. Glycaemic variability, rather than HbA1c, may be more important in predicting the micro and macrovascular complications of diabetes as well as the immune impairment/susceptibility to respiratory virus infection seen in patients with DM [1–3]. Glycaemic variability is most frequently measured by continuous glucose monitors (CGMs). However, in the absence of subsidized funding the costs of CGMs can be prohibitive, meaning that glycaemic variability is not routinely measured in all patients with DM [4, 5].

1,5 anhydroglucitol (1,5-AG) has been studied as an alternative biomarker for glycaemic variability in the absence of CGM data [6–13]. Naturally occurring as a 1-deoxy form of glucose in food, 1,5-AG circulates around the human body and is balanced by urinary excretion [13]. In healthy individuals, 99.9% of 1,5-AG is reabsorbed in renal tubules back into the blood to maintain healthy levels. However, increased levels of filtered glucose in hyperglycaemic excursions competes for tubular reabsorption, leading to decreased circulating 1,5-AG levels and increased 1,5-AG urinary loss [13]. Accordingly, lower serum levels of 1,5-AG have shown potential as a marker for postprandial hyperglycaemic events in both type 1 [14] and type 2 DM [7]. 1,5-AG levels were also more sensitive than fructosamine [11] and HbA1c [10, 11, 15] in reflecting glycaemia improvements with various glycaemic controls as early as 2 weeks. Accordingly, this marker has been used clinically for the monitoring of DM in Japan for decades [16]. However, 1,5-AG is not widely used elsewhere in the clinical management of diabetes. This may be due to suggestions that 1,5-AG is only correlated with glycaemic excursions in well controlled DM (i.e. HbA1c levels < 8.0%) [6, 7, 10, 15]. Moreover, the ability of 1,5-AG to predict more recent markers of glycaemic variability, such as the glycaemia risk index (GRI) has yet to be shown. The GRI is a novel composite metric assessing overall glycemic risk derived from CGM data [17]. The GRI prioritizes hypoglycemia over hyperglycemia and places more emphasis on extreme cases of either condition [17]. Importantly, the GRI was developed to provide a simple but complete picture of glycaemic control that reflected diabetologists’ ranking of the glycemic quality of CGM tracings of people with DM [18]. Accordingly, higher GRI has been associated with worse quality of life, increased diabetes-related stress and reduced satisfaction with treatment [19].

Here, we aim to determine whether assessing 1,5-AG, in addition to HbA1c, is of any potential clinical utility to the management of DM for patients, in particular in terms of assessment of GRI and anti-viral immune function.

## Materials and methods

### Patient recruitment

#### Australian cohort

A cohort of 29 patients clinically diagnosed with type 1 DM were recruited. These individuals were a subset of a previously described cohort of 72 patients with diabetes mellitus (T1D and T2D) [20]. Inclusion criteria were 18–60 years of age, not pregnant at the time of study, non-smokers, minimum diabetes mellitus duration of two years and no known immune disease requiring immunosuppressants. At recruitment, blood and clinical data were collected as well as two weeks’ of CGM data. This study was approved by Mater Research Ethics Committee (HREC/MML/55151 V2) and UQ Ethics Committee (2019/HE002522). All methods were performed in accordance with institutional guidelines and regulations. Written consent was obtained from all study participants.

#### French cohort

A cohort of 49 patients aged of 18 years old or more with clinically diagnosed type 1 DM were recruited. Patients had been living with type 1 DM for at least 10 years at the time of study recruitment and were using a FreeStyle Libre CGM. Exclusion criteria were type 2 DM, corticotherapy, cancer, pregnancy and breastfeeding and complicated proliferative retinopathy. CGM metrics used for the analysis were assessed using CGM data from the last 30 days before inclusion visit. The study was approved by the ethics committee (CPP Est I, Dijon, France), and each participant gave written informed consent prior to participation. The trial is registered at clinicaltrials.gov as NCT03821753.

Across both cohorts the following definitions were used: time spent very low (glucose levels below 54 mg/dL), time spent low (glucose levels between 54 mg/dL and 70 mg/dL), time spent high (glucose levels between 180 mg/dL − 250 mg/dL) and time spent very high (glucose levels above 250 mg/dL). GRI was calculated as (3.0 × VLow) + (2.4 × Low) + (1.6 × VHigh) + (0.8 × High).

### Human plasma and PBMC isolation

Approximately 10mL of whole peripheral blood was collected in BD Vacutainer^®^ EDTA tubes from both patient cohorts. Plasma was collected from both cohorts by centrifugation and stored at -80 ◦C until use. Peripheral blood mononuclear cells (PBMCs) were isolated with Lymphoprep (STEMCELL, Canada) from the Australian cohort according to manufacturer’s instructions. Isolated PBMCs were subsequently frozen down in Fetal Calf Serum (FCS) (Gibco) containing 10% DMSO (Sigma-Aldrich) and stored at -80 ◦C until use.

### 1,5 Anhydroglucitol (1,5-AG) levels

To quantify plasma 1,5-AG levels, the GLYCOMARK test kit (Nippon Kayaku Inc, Tokyo, JP) was used as per manufacturer’s instructions [11, 21]. In short, plasma samples were all pre-treated with glucokinase to remove any glucose present. 1,5-AG levels were detected by the addition of enzyme pyranose oxidase, forming hydrogen peroxide. Samples were subsequently visualized by the addition of peroxidase and analyzed at 546/700nm on the Hitachi 917 automatic analyser (Roche Diagnostics Corporation, Indianapolis, IN). For reference, 1,5-AG in males and females without diabetes are reported to range from 15.8 to 52.6 µg/ml and 14.3–48.0 µg/ml respectively [22].

### Influenza virus culture

HKx31 (H3N2) virus stocks were prepared in embryonated chicken eggs. As previously published [23], infectious viral titres were determined by MDCK plaque assays. The use of embryonated chicken eggs was approved by the University of Queensland Animal Ethics Committee (AE000089).

### T cell stimulation and intracellular cytokine staining (ICS) on human samples

To investigate the effect of glycaemic variability on CD4 + T cell cytokines ex vivo, PBMCs from donors in the Australian cohort were stimulated with either (i) HKx31 (multiplicity of infection 10), (ii) 25ng/mL PMA and 1 µg Ionomycin (Sigma Aldrich) (iii) RPMI (Gibco) with 10% FCS (Gibco) or (iv) CD3/CD28 beads for 18 h in the presence of anti-human CD107a (0.4 µg/mL; BioLegend; H4A3), BD GolgiStop (BD Biosciences) and GoliPlug (BD Biosciences) as previously [3, 20]. T cells were subsequently washed and stained for CD3, CD4 and CD8. T cells were then washed, fixed, and permeabilised using the Cytofix/Cytoperm Fixation/Permeabilization kit (BD Biosciences) and stained with anti-MIP1β, anti-IFNγ and anti-TNF [3, 20]. Cells were run on the LSRFortessa (BD Biosciences) and analysed using FlowJo v10.8 (BD Biosciences).

### Statistical analysis and data availability

A. Mann-Whitney test, Student t-test, Pearson correlation coefficient, paired t-test, Wilcoxon test or Fisher’s exact test on patient data was performed using GraphPad Prism software (version 9.3.1) (Dotmatics, CA, USA) where normality was assessed with a Shapiro-Wilk normality test.

Multiple Linear Regression (MLR) was performed using MATLAB (R2022a) and the code is available 10.48610/d582ee0.

Regression tree analysis was performed using MATLAB (R2022a) and the Classification and Regression Trees (CART) method to model the relationship between the predictors and the response variable. To ensure the robustness and generalizability of the model, 10-fold cross-validation was employed. In this procedure, the data was randomly partitioned into ten equal subsets, and the model was trained on nine subsets while being validated on the remaining one, in an iterative process. The performance of a single tree was then evaluated using the average mean squared error of all model estimates against their corresponding true values of the outcome variable across all validation sets. The code is available at 10.48610/d582ee0.

Patients with a matching HbA1c but differences in 1,5-AG for immune analysis were identified using MedCalc version 22.030 with the following parameters: HbA1c (< 1% difference between groups; HbA1c < 10), age (< 40 years difference between groups) and sex (identical between groups).

## Results

### Multiple Linear regression of 1,5-AG, HbA1c and CGM data

To determine the potential role of 1,5-AG in monitoring glycaemic variability we collected data from 29 Australian DM patients and 49 French DM patients for whom CGM data was available. Patients had a median age of 32.45 years, a median BMI of 24.72 kg/m^2^, a median 1,5-AG of 3.5 µg/mL and a median HbA1c of 7.4% (Supplementary Table 1).

**Fig. 1:**
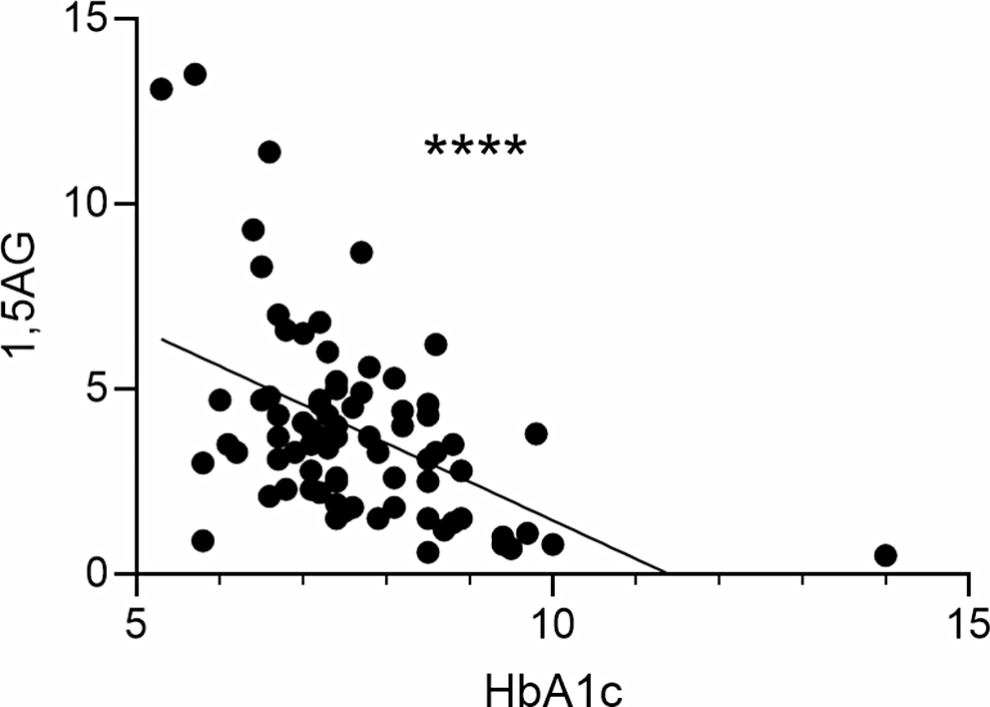
HbA1c and 1, 5-AG are highly correlated in patients with type 1 diabetes. Data was obtained from 49 type 1 diabetes patients recruited from France and 29 recruited from Australia. Statistical significance was determined with Person’s correlation coefficient where *p* < 0.0001(****)

A MLR was subsequently performed to assess the association between 1,5-AG/HbA1c and percentage time in range, percentage time very high, percentage time low and GRI. Percentage time very low was not included in the analysis as it failed the assumption of residual homoscedasticity (data not shown). In light of the potential confounding effects of age, sex, BMI and site these factors were included as covariates. Importantly, and consistent with prior reports [11], 1,5-AG and HbA1c were highly correlated (Fig. 1). Therefore, including the variables HbA1c and 1,5-AG in the same MLR would violate the assumption of limited multicollinearity upon which a MLR is based [24]. Accordingly, two separate MLR models for 1,5-AG and HbA1c were created (Supplementary Tables 2 and 3). Both 1,5-AG and HbA1c levels had a significant association with percentage time in range, percentage time very high, percentage time high, percentage time low and GRI when controlling for the aforementioned covariates (Supplementary Tables 2 and 3).

### Regression tree analysis of GRI

**Fig. 2:**
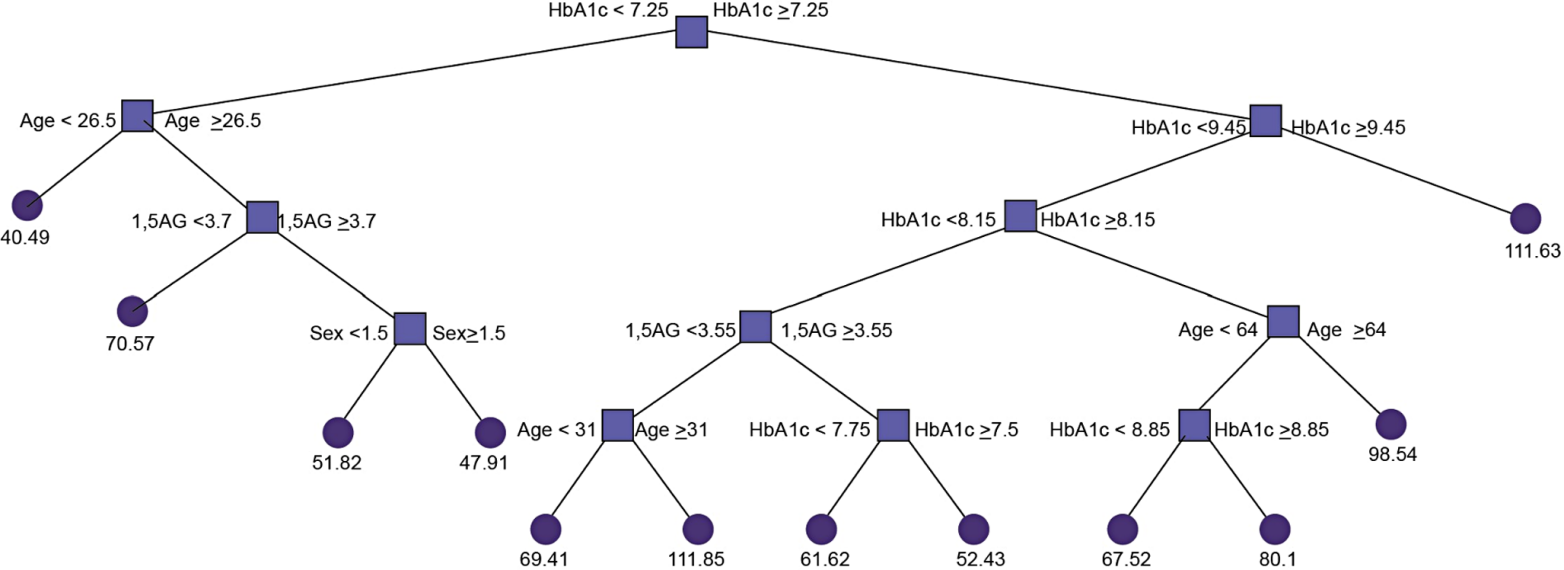
Regression tree for GRI incorporating age, sex, site, HbA1c and 1,5-AG. Numbers at the end of each node indicate the predicted GRI

**Fig. 3:**
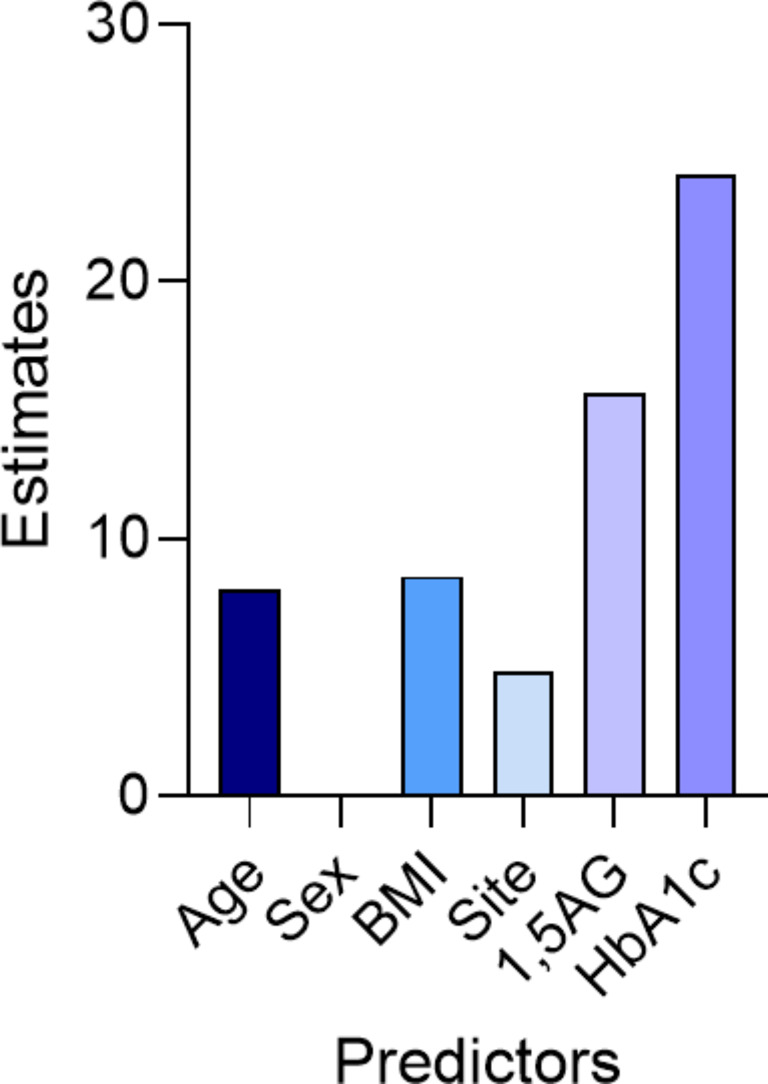
Feature importance in model 1 (HbA1c, 1,5-AG, age, sex, site and BMI)

The above data indicate a strong linear relationship between both 1,5-AG and HbA1c and CGM-derived measures of glycaemic variability. However, these data do not indicate whether the addition of 1,5-AG provides increased insight into a patient’s glycaemic variability compared to measuring HbA1c alone. To answer this question, we turn to methods in machine learning. Specifically, we developed a regression tree for a patient’s GRI (Fig. 2). A regression tree is a predictive model that splits data into progressively smaller groups based on input features. At each split, it chooses a feature and value that best separates the data to minimize prediction errors. Such a model can thus be used clinically in a predictive context, where the flowchart can be followed and the GRI of a patient without a CGM can be predicted based on the features of the tree. However, to develop such a model for robust GRI prediction in the clinic it would be necessary to divide data into a training and test set, which is not possible with the n number of the present study. Rather, here we sought to use a regression tree to simply determine if 1,5-AG offers any additional predictive power on the estimated value of GRI over HbA1c alone. In this context, it is appropriate to test the validity of the model using 10-fold cross-validation, with a 90% training set and 10% validation set. With this in mind we created three different regression trees – one including just HbA1c (in addition to age, sex, BMI and site), one including just 1,5-AG (in addition to age, sex, BMI and site) and one including both HbA1c and 1,5-AG (in addition to age, sex, BMI and site) (Table 1). To compare the performance across each tree, the mean squared error is calculated for GRI prediction on each fold iteration and averaged over all folds (Table 1). In this context, a lower average mean square indicates improved GRI prediction (Table 1). Consistent with an additive role of 1,5-AG and HbA1c in diabetes management the best performing model was Model 1, which included age, sex, site, BMI, HbA1c and 1,5-AG (Table 1). Surprisingly, model 2 (1,5-AG, age, sex, site and BMI) had a lower average mean square error than Model 3 (HbA1c, age, sex, site and BMI) (Table 1). To explore these data further we assessed the importance of each feature (e.g. HbA1c, age, sex etc.) in Model 1 using the CART method [25]. Figure 3 shows that HbA1c and 1,5-AG were the features with the highest predictive value, with HbA1c classified at a higher importance over 1,5-AG.

**Table 1:** Regression tree analysis with both HbA1c and 1,5-AG, 1,5-AG alone or HbA1c alone

	Model 1 – HbA1c and 1,5-AG	Model 2–1,5-AG	Model 3 – HbA1c
Variables	Age, sex, BMI, site, HbA1c, 1,5-AG	Age, sex, BMI, site, 1,5-AG	Age, sex, BMI, site, HbA1c
Average mean square error	139.62	143.43	290.32

**Fig. 4:**
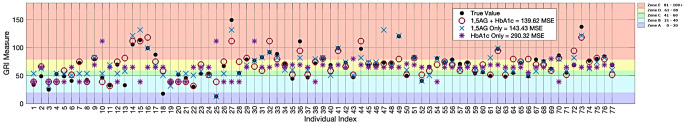
1,5-AG provides the greatest predictive value for individuals with a GRI is Zone A and E. Zones were defined according to [26]. True GRI values are shown in black. Predicted values from Model 1 (1,5-AG, HbA1c, BMI, Age, Site and Sex) are shown in red circles. Predicted values from Model 2 (1,5-AG, BMI, Age, Site and Sex) are shown with a blue cross. Predicted values from Model 3 (HbAc1, BMI, Age, Site and Sex) are shown with a purple asterisk. Individual Index refers to the donor ID number. MSE = Average Mean Square Error

These data (Fig. 3) contrast with those shown in Table 1 where the model using 1,5-AG alone (Model 2) better predicts GRI compared to the HbA1c alone model (Model 3). To explore this discrepancy further we examined the predictive capacity of the three different models across the range of GRIs recorded herein (Fig. 4). Here, we show that Model 3 (HbA1c alone) is best able to predict the values of GRI from individuals whose GRI falls in zone B (21–40), zone C (41–60) and zone D (61–80) [26]. This reflects the majority of the individuals in the cohort and is hence in agreement with the greater importance of HbA1c as a feature (Fig. 3). In contrast, 1,5-AG is able to more accurately predict values of GRI that fall in zone A (0–20) and zone E (81+)^28^ (Fig. 4), allowing 1,5-AG to contribute significantly to the decrease in mean sqaured error of the model (Table 1). Taken together, these data indicate that 1,5-AG provides additional predictive information to determine a patient’s GRI above that offered by HbA1c alone. The inclusion of 1,5-AG is particularly important for individuals with a GRI that falls within zone A or zone E.

### 1,5-AG and the CD4 + T cell cytokine responses to ex vivo stimulation with influenza virus in individuals with a matched HbA1c

Glycaemic variability, rather than just HbA1c, is important in predicting the micro and macrovascular complications of diabetes [1] as well as the immune impairment/susceptibility to respiratory virus infection seen in patients with DM [1–3]. We thus sought to explore if 1,5-AG provided any additional information (above that of HbA1c) to predict the anti-viral immune response in patients with DM. As described above, this analysis could not be performed using MLR with HbA1c and 1,5-AG as covariates as these values are highly correlated (Fig. 1) and thus violate the assumptions of multiple linear regression. Instead, individuals from the Australian cohort were classified as having a ‘low’ or ‘high’ 1,5-AG. There is currently no accepted clinical definition of a ‘low’ or ‘high’ 1,5-AG for patients with DM. Rather individuals were classified as having a ‘low’ 1,5-AG if the 1,5-AG levels were lower than the range observed for 368 patients with DM (i.e. 1,5-AG *≤* 1.1)^29^. This categorical grouping allowed us to match individuals with ‘low’ and ‘high’ HbA1c based on their HbA1c (< 1% difference; HbA1c < 10), age (< 40 years difference) and sex (identical) and assess their ex vivo immune response to influenza virus. Supplementary Table 4 demonstrates that 7 matched individuals were selected who had a significant difference in 1,5-AG levels but no significant difference in HbA1c, age, sex or BMI.

**Fig. 5:**
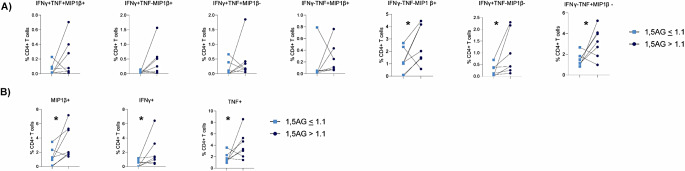
Low 1,5-AG is associated with reduced CD4 + T cell cytokine production in response to ex vivo stimulation with influenza virus. (**A**) Polyfunctional analysis of CD4 + T cells. (**B**) Total percentage of MIP1β, IFNγ or TNFα positive CD4 + T cells. Statistical significance was assessed using a paired t-test (normally distributed data) or a Wilcoxon test (not normally distributed data). *p < 0.05 1,5-AG *≤* 1.1 is shown in light blue squares whilst 1,5-AG > 1.1 is shown in dark blue circles

**Fig. 6:**
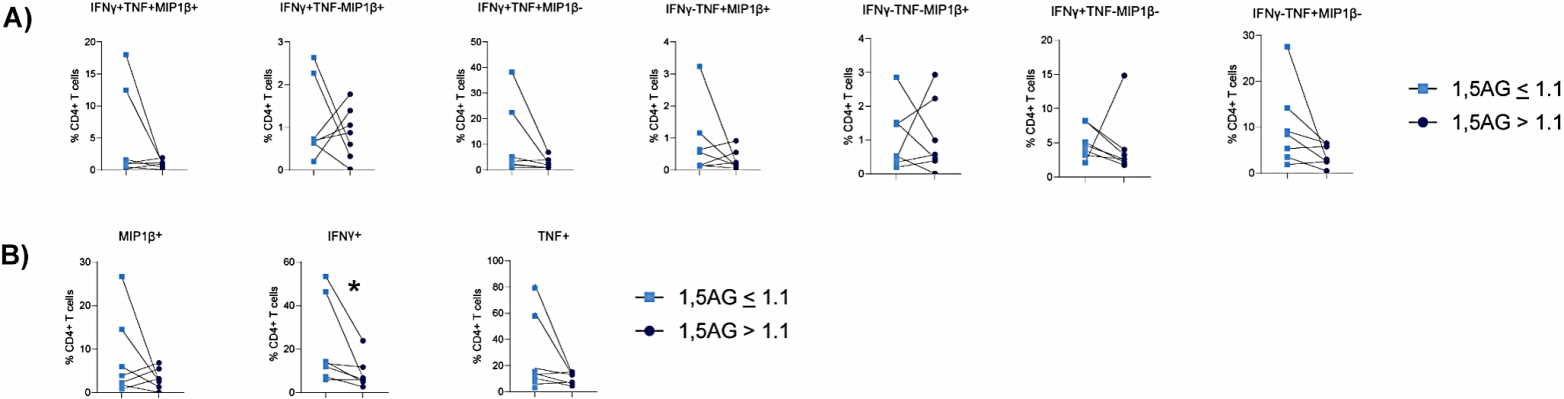
Low 1,5-AG is not associated with reduced CD4 + T cell cytokine production in response to ex vivo stimulation with PMA/I. (**A**) Polyfunctional analysis of CD4 + T cells. (**B**) Total percentage of MIP1β, IFNγ or TNFα positive CD4 + T cells. Statistical significance was assessed using a paired t-test (normally distributed data) or a Wilcoxon test (not normally distributed data). *p < 0.05

**Fig. 7:**
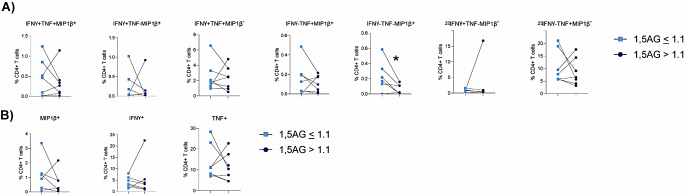
Low 1,5-AG is not associated with reduced CD4 + T cell cytokine production in response to ex vivo stimulation with CD3/CD28 magnetic beads. (**A**) Polyfunctional analysis of CD4 + T cells. (**B**) Total percentage of MIP1β, IFNγ or TNFα positive CD4 + T cells. Statistical significance was assessed using a paired t-test (normally distributed data) or a Wilcoxon test (not normally distributed data). *p < 0.05

To evaluate if 1,5-AG could provide any insight into T cell function, in the presence of matched HbA1c, the cytokine response of CD4 + T cells to ex vivo stimulation with influenza virus in the 7 matched donors was assessed (Fig. 5). Cytokine production by CD4 + T cells is an essential part of the anti-viral immune response as CD4 + T cell derived cytokines play a key role in activating CD8 + T cells and B cells, whilst also recruiting and activating innate antigen presenting cells such as dendritic cells. HbA1c matched individuals with a lower 1,5-AG had a significantly lower percentage of CD4 + IFNγ^+^TNF^−^MIP1β^−^, CD4 + IFNγ^−^TNF^+^MIP1β^−^ and CD4 + IFNγ^−^TNF^−^MIP1β^+^ cells in response to influenza virus stimulation (Fig. 5). Consistent with these data, matched individuals with type 1 DM and a lower 1,5-AG also had a significantly lower percentage of total CD4 + IFNγ^+^, CD4 + TNF^+^ and CD4 + MIP1β^+^ cells in response to influenza virus stimulation (Fig. 5). These differences were specific to influenza virus stimulation, as the 7 matched individuals with a lower 1,5-AG did not have a significantly lower CD4 + T cell cytokine positive cells in response to stimulation with PMA/I (Fig. 6) or CD3/CD28 beads (Fig. 7). Taken together, these data indicate that 1,5-AG can provide additional insight as to CD4 + T cell function above that which is provided by HbA1c alone. Specifically, these data suggest that low 1,5-AG can reduce the number of CD4 + T cell cytokine producing cells in response to influenza virus.

## Discussion

The availability of CGM data has provided important insights into the glycaemic variability experienced by patients living with diabetes. Understanding this glycaemic variability is essential to help predict the micro and macrovascular complications of diabetes as well as the immune impairment/susceptibility to respiratory virus infection seen in patients with DM [1, 2]. Unfortunately, at present the costs associated with CGMs can be prohibitive, meaning that glycaemic variability is not routinely measured in all patients with DM [4, 5]. Here, we explore whether the measurement of plasma 1,5-AG, in addition to routine measurements of HbA1c, can provide additional insights into glycaemic variability in patients living with diabetes.

GRI is a composite measure of glycaemic variability which captures both hyperglycemia and hypoglycemia events, although hypoglycaemia events are weighted more heavily [17]. GRI has become popular to calculate for the management of DM as it reflects clinical priorities, patient centred outcomes and is associated with numerous diabetic complications [18, 19, 28, 29]. As GRI is a weighted calculation based upon time spent very low (glucose levels below 54 mg/dL), time spent low (glucose levels between 54 mg/dL and 70 mg/dL), time spent high (glucose levels between 180 mg/dL − 250 mg/dL) and time spent very high (glucose levels above 250 mg/dL) its calculation is dependent upon the availability of CGM data. Here, we provide the first evidence that serum 1,5-AG levels are strongly associated with the GRI in patients living with type 1 DM. Consistent with previous reports [30], a patient’s HbA1c was also associated with the GRI. Therefore, to determine 1,5-AG provides increased insight into a patient’s glycaemic variability compared to measuring HbA1c alone a regression tree for GRI was developed. Regression trees are highly useful for such analysis as they are easy to visualize and interpret and they are capable of capturing non-linear relationships between features and the target variable. Whilst the regression tree developed herein requires further validation prior to use in the clinic as tool to predict GRI in people without CGM data, it highlights the value of measuring 1,5-AG to predict a patient’s GRI. The inclusion of 1,5-AG in this prediction was particularly important for individuals who had very high or very low GRI.

We have previously shown that glycaemic variability (as detected by the coefficient of variation derived from CGM data) in participants living with type 1 DM with matched HbA1c is associated with a reduced percentage of cytokine producing T-cells in response to ex vivo stimulation with influenza virus [3]. It is hypothesised that this may help explain the increased risk of severe respiratory virus disease experienced by patients with DM [31–34], in particular those with high glycaemic variability [34–37]. Here, we show that in the absence of CGM data 1,5-AG levels are associated with differences in the ex vivo response of CD4 + T cells to influenza virus in individuals with matched HbA1c. Specifically, we showed that matched individuals with a lower 1,5-AG also had a significantly lower percentage of CD4 + IFNγ^+^, CD4 + TNF^+^ and CD4 + MIP1β^+^ cells in response to influenza virus stimulation. Interestingly, this same phenotype was not observed following stimulation with PMA/I and CD3/CD28 beads. This is in contrast to prior observations that an elevated HbA1c was associated with a reduced ex vivo T cell response to both influenza virus and CD3/CD28 beads [20]. CD3/CD28 beads stimulate the T cell response via the T cell receptor (TCR) complex whilst PMA/I facilitates the transport of calcium across the plasma membrane resulting in the activation MAPK [20]. The phenotypic difference observed herein following these different stimulations may suggest that 1,5-AG best reflects differences in antigen specific stimulation (e.g. via affecting antigen processing/presentation). Indeed, it is interesting to note that glycaemic variability is associated with increased oxidative stress [2] and that oxidative stress is linked to a reduced capacity of antigen presenting cells to process antigens and to initiate a primary T cell response [38].

The present study was subject to several limitations. Firstly, our study has focused on individuals living with Type 1 DM. It remains to be determined if 1,5-AG could similarly be used to reflect GRI and immune function in individuals with Type 2 DM. This is particularly pertinent to determine given that CGMs are less frequently available to this patient group [39]. The limited n number of the present study also restricted the broader predictive capacity of the regression tree for GRI developed herein, although it must be noted that developing a broadly predictive model for GRI was not the primary goal of this study. Similarly, only a limited number of samples were available for immune analysis as patients had to be matched on HbA1c, age and sex. This was necessary given the prior described effects of these co-variables on the T cell response [20, 40, 41]. There is also not sufficient evidence to state whether these changes in the ex vivo response to influenza virus contribute to the increased severity of respiratory virus infections in individuals with diabetes and high glycaemic variability [34–37]. Rather, our data suggest that 1,5-AG provides an additional insight into a patient’s T cell response beyond that which is available by measuring HbA1c alone.

Several steps are required prior to the widespread use of 1,5-AG for the clinical management of diabetes. Firstly, should 1,5AG indeed have clinical utility in assessing glycaemic variability, the assessment of 1,5-AG needs to be affordable and broadly available. It is also necessary to determine a ‘target’ 1,5-AG value for patients living with DM. Currently, the range of 1,5-AG reported in patients living with DM is broad, ranging from 1.1 µg/mL – 25.6 µg/mL [27]. A target 1,5-AG value is thus required for patients living with diabetes which reflects the lowest risk for glycaemic variability and diabetic complications.

## Conclusions

Whilst further progress is necessary prior to the widespread, routine clinical measurement of 1,5-AG, these data show that monitoring 1,5-AG provides additional information compared to just monitoring a patient’s HbA1c alone and could be particularly important for individuals who do not have access to a CGM.

## Electronic supplementary material

Below is the link to the electronic supplementary material.

Supplementary Material 1

## Data Availability

All data and materials are available from the corresponding upon request.
