# Supplementary material for: Measurement of serum 1,5-AG provides insights for diabetes management and the anti-viral immune response
Source: Cell Mol Life Sci. 2025 Feb 6;82(1):71. doi: 10.1007/s00018-024-05568-7 (PMC11803061; doi:10.1007/s00018-024-05568-7)
Supplement: Supplementary file 1 — Supplementary Material 1 [file 18_2024_5568_MOESM1_ESM.docx]

**Supplementary Table 1: Summary Statistics of patients with CGM data available**

| Variable | Summary Statistics | Range |
| --- | --- | --- |
| Age (years) | Median: 32.45 | Min: 18.5  Max: 68 |
| Sex | Female: 44/78 [56.4]  Male: 34/78 [43.6] |  |
| BMI (kg/m^2^) | Median: 24.72 | Min:17.8  Max: 42.6 |
| Site | Australia: 29/78 [37.2%]  France: 49/78 [62.8%] |  |
| 1,5-AG (µg/mL) | Median: 3.5 | Min: 0.5  Max: 13.5 |
| HbA1c (%) | Median:7.4 | Min:5.3  Max: 14 |
| Percentage time in range (TIR) | Median: 45.7 | Min:1  Max: 90 |
| Percentage time high | Median: 29.83 | Min:6  Max: 56.25 |
| Percentage time very high | Median: 13.9 | Min:0  Max: 88 |
| Percentage time low | Median: 3.7 | Min: 0  Max: 21.43 |
| Percentage time very low | Median: 0 | Min:1  Max: 32.93 |
| Glycaemia risk index (GRI) | Median: 69.15 | Min:12.8  Max: 149.6 |

| **Model Outcome Variable** | **Age** | **p-value** | **BMI** | **p-value** | **Sex** | **p-value** | **Site** | **P-value** | **1,5-AG** | **p-value** | **R-squared** |
| --- | --- | --- | --- | --- | --- | --- | --- | --- | --- | --- | --- |
| Percentage time in range | 0.062 | 0.66 | -0.54 | 0.19 | 2.8 | 0.45 | -10.77 | **0.02** | 3.69 | **8.56E-07** | 0.358 |
| Percentage time high | -0.119 | 0.33 | 0.5 | 0.16 | -2.67 | 0.41 | -5.52 | 0.15 | -2.61 | **3.91E-05** | 0.294 |
| Percentage time very high | 0.051 | 0.59 | 0.002 | 0.99 | -0.79 | 0.76 | 9.7 | **1.60E-03** | -1.58 | **1.18E-03** | 0.289 |
| Percentage time low | 0.005 | 0.9 | 0.086 | 0.43 | 1.05 | 0.3 | 4.09 | **7.25E-04** | 0.45 | **1.57E-02** | 0.251 |
| GRI | -0.131 | 0.54 | 0.86 | 0.16 | -3.51 | 0.53 | 16.5 | **0.01** | -4.07 | **1.17E-04** | 0.275 |

**Supplementary Table 2: MLR for 1,5-AG and CGM data where Age, BMI, Sex, Site and 1,5-AG were independent variables**

**Supplementary Table 3: MLR for HbA1c and CGM data where Age, BMI, Sex, Site and HbA1c were independent variables**

| **Model Outcome Variable** | **Age** | **p-value** | **BMI** | **p-value** | **Sex** | **p-value** | **Site** | **P-value** | **HbA1c** | **p-value** | **R-squared** |
| --- | --- | --- | --- | --- | --- | --- | --- | --- | --- | --- | --- |
| Percentage time in range | -0.034 | 0.75 | -0.16 | 0.6 | 0.11 | 0.97 | -8.91 | **7.82E-03** | -10.61 | **4.77E-15** | 0.627 |
| Percentage time high | -0.018 | 0.79 | 0.07 | 0.73 | -0.65 | 0.71 | -7.13 | **9.16E-04** | 14.281 | **1.46E-22** | 0.759 |
| Percentage time very high | 0.064 | 0.51 | -0.03 | 0.91 | -0.08 | 0.97 | 8.79 | **4.36E-03** | 3.57 | **6.40E-04** | 0.3 |
| Percentage time low | -0.007 | 0.84 | 0.14 | 0.16 | 0.92 | 0.31 | 4.53 | **4.65E-05** | -5.14 | **2.32E-06** | 0.401 |
| GRI | -0.005 | 0.98 | 0.37 | 0.48 | 0.23 | 0.96 | 14.92 | **9.69E-03** | 11.71 | **1.80E-08** | 0.439 |

**Supplementary Table 4 - Matched individuals from the Australian cohort of patients with type 1 DM**

|  | **‘Low’ 1,5-AG (n = 7)**  **Mean (±SD)** | **‘High’ 1,5-AG (n = 7)**  **Mean (±SD)** | **P-value** |
| --- | --- | --- | --- |
| **HbA1c (%)** | 8.6 (±1.27) | 8.114 (±0.9788) | P = 0.07^1^ |
| **Age (years)** | 32.31 (±13.58) | 32.39 (±14.09) | P = .99^2^ |
| **Sex** | Female: 4/7 [57%]  Male: 3/7 [43%] | Female: 4/7 [57%]  Male: 3/7 [43%] | P = 1^3^ |
| **BMI** | 28 (±6.9) | 28.1 (±6.9) | P=.98^2^ |
| **1,5-AG (µg/mL)** | 0.93 (±.21) | 4 (±2.1) | P = 0.003^2^ |

**^1.^**Mann-Whitney test ^2.^ Student t-test ^3.^ Fisher’s exact test
